# Calcium, *ARMS2* Genotype, and *Chlamydia Pneumoniae* Infection in Early Age-Related Macular Degeneration: a Multivariate Analysis from the Nagahama Study

**DOI:** 10.1038/srep09345

**Published:** 2015-03-20

**Authors:** Isao Nakata, Kenji Yamashiro, Takahisa Kawaguchi, Hideo Nakanishi, Yumiko Akagi-Kurashige, Masahiro Miyake, Akitaka Tsujikawa, Ryo Yamada, Fumihiko Matsuda, Nagahisa Yoshimura, Nagahama Study Group

**Affiliations:** 1 Department of Ophthalmology, Kyoto University Graduate School of Medicine, Kyoto, Japan; 2 Center for Genomic Medicine/Inserm U.852, Kyoto University Graduate School of Medicine, Kyoto, Japan

## Abstract

Although various risk factors have been identified for the development of age-related macular degeneration (AMD), risk factors of early AMD have been relatively under studied. We aimed to investigate AMD risk factors by evaluating multiple factors in association with large drusen, an important component of AMD, simultaneously. In a community-based cross-sectional survey in Japan, 971 large drusen cases and 3,209 controls were compared for 65 variables, including systemic, environmental, and genetic factors. The association and the effect size of each factor were evaluated with logistic regression analysis using a backward-elimination approach. Multivariate analyses identified a significant association in serum calcium level (odds ratio [OR] = 0.932, *P* = 1.05 × 10^−3^), *ARMS2* A69S (rs10490924) genotype (OR = 1.046, *P* < 0.001), *Chlamydia pneumoniae* IgG (OR = 1.020, *P* = 0.0440), and age (OR = 1.013, *P* < 0.001) for large drusen. Hypocalcemia was observed in 7.2% of large drusen cases and in 5.5% of controls (*P* = 0.0490). *C. pneumoniae* infections was more frequent in large drusen cases (56.4%) than in conrols (51.7%, *P* = 0.00956). These results suggest that calcium, *ARMS2* genotype, *C. pneumonia* infection, and age are significant factors in the development of the early stages of AMD.

Age-related macular degeneration (AMD) is the leading cause of blindness among the elderly population in developed countries^1^. Early signs of AMD are characterized by drusen, small extracellular deposits between the retinal pigment epithelium (RPE) and the inner collagenous layer of Bruch's membrane, or by pigment abnormalities in the RPE in the macula^1^. Although visual symptoms are generally inconspicuous in the early stages of AMD^2^, the presence of early signs is highly associated with the progression to advanced AMD, in which visual function is severely damaged^3^. Because the treatment options for advanced AMD is still limited and the associated economic burden is increasing^4^, studying and preventing disease progression during the early stages of AMD are of increasing importance.

Age, smoking, and mutations in several genes are the most consistently identified risk factors for AMD^1^. These associations have been confirmed in populations across the globe, including Western and Asian countries^5^,^6^,^7^,^8^,^9^. To date, various other factors have been suggested to increase the risk of AMD^10^,^11^,^12^,^13^. However, the evidence and strength of these associations remain controversial^14^,^15^,^16^. The pathophysiology of AMD is still poorly understood and considered to be a constellation of diseases of varying etiologies.

Identifying the “true” risk factors for complex diseases such as AMD is a daunting task because multiple factors, including systemic, environmental, and genetic factors, contribute to disease liability with a small effect size. In fact, by analyzing a large number of subjects, recent genetic studies for complex diseases revealed several loci with smaller effect sizes, such as odds ratios (ORs) of 1.08–1.16^17^. To identify risk factors of complex disease and to determine the significance of each factor, a simultaneous analysis of multiple factors using a large number of subjects is necessary. In the present study, we sought to simultaneously investigate multiple risk factors for large drusen, an important early sign of AMD that has been shown in many longitudinal studies to be predictive of incident AMD advanced^3^,^18^, using a relatively large number of Japanese adults.

## Results

### Multivariate analysis for drusen

The distributions of predominant characteristics according to 971 large drusen cases and 3,209 controls are shown in Table 1. Study cases were significantly older than controls (65.1 ± 5.9 and 61.7 ± 6.5 years, respectively; *P* < 0.001). In the genetic analyses, a significant association was found between *ARMS2* A69S (rs10490924) and drusen; the frequency of the minor allele T, which is known as a risk allele for developing advanced AMD, was significantly higher in cases than in controls (*P* < 0.001). Conversely, *CFH* Y402H (rs1061170) and *CFH* I62V (rs800292) did not show a significant association with drusen (*P* > 0.05).

**Figure 1:**
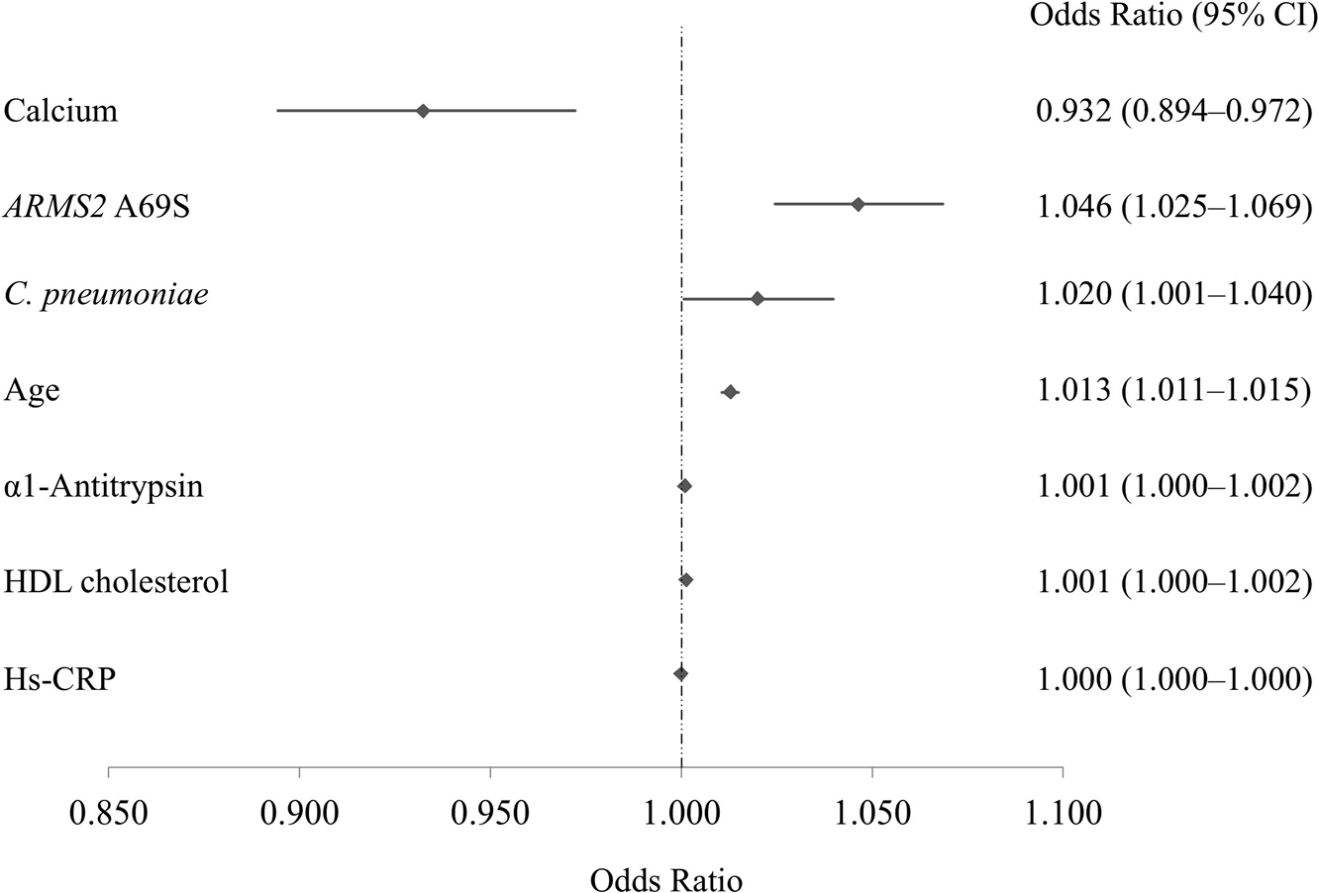
The relative strength of the significant factors for large drusen that showed a significant association in the final multivariate analysis. Serum calcium level had the strongest effect on the development of large drusen; the other factors were *ARMS2* A69S (rs10490924) genotype, *Chlamydia* *pneumoniae* IgG, and age. High-density lipoprotein (HDL) cholesterol, α1-antitrypsin, and high-sensitivity C-reactive protein (hs-CRP) had limited effect on disease development with odds ratio between 1.000 and 1.001. A diamond represents the point estimate of odds ratio for each factor with a horizontal line of the 95% confidence interval (CI).

Each subject was tested for 65 factors, including 60 systemic factors, smoking status, Brinkman index, and the genetic factors *ARMS2* A69S, *CFH* Y402H, and *CFH* I62V (Table S1). After excluding two correlated variables (e.g., *C. pneumoniae* IgA and C-telopeptides crosslinks in lieu of the *C. pneumoniae* IgG and N-telopeptides crosslinks, respectively), 63 systemic factors were compared between cases and controls using univariate analyses. With this screening, a total of 29 factors, including age, systolic blood pressure, and genetic factors *ARMS2* A69S and *CFH* I62V, showed a potential association with drusen (*P* < 0.25; Table S2). Stepwise selection using a backward-elimination approach was performed for the 29 factors after adding five more previously reported AMD risk factors (BMI^11^, HDL cholesterol^12^, hs-CRP^13^, smoking status, and *CFH* Y402H genotype). Table 2 summarizes the final multivariate analysis of all candidate predictor variables selected by stepwise analysis. Of the nine candidates, seven showed a significant association with drusen (*P* < 0.05): age, *ARMS2* A69S genotype, serum calcium, HDL cholesterol, α1-antitrypsin (AAT), hs-CRP, and *C.* *pneumoniae* IgG. Figure 1 shows the relative strength of each significant factor for drusen. We found that four of seven factors have a strong effect on the development of drusen: serum calcium level (OR 0.932, 95% confidential interval [CI] 0.894–0.972), *ARMS2* A69S (rs10490924) genotype (OR 1.046, 95%CI 1.025–1.069), *C.* *pneumoniae* IgG (OR 1.020, 95%CI 1.001–1.040), and age (OR 1.013, 95%CI 1.011–1.015).

### Calcium level distribution and *C. pneumoniae* infection

Serum calcium levels in drusen cases were significantly lower than controls (9.05 and 9.11 mg/dL, respectively; *P* < 0.001). When applying the normal range of serum calcium (8.6–10.3 mg/dL), hypocalcemia (calcium level <8.6 mg/dL) was more frequent in the large drusen cases than in controls; 7.2% in drusen cases and 5.5% in controls, respectively (*P* = 0.0490; Table 3).

*C. pneumoniae* IgG levels were significantly elevated in drusen cases compared with controls (1.30 and 1.23, respectively; *P* < 0.001). We also confirmed a higher frequency of *C. pneumoniae* infections in drusen cases (56.4%) compared with controls (51.7%; *P* = 0.00956) by measuring serum reactivity according to the standard cutoff index for the IgG titer: <0.9, negative; 0.9 to 1.1, equivocal; and >1.1, positive (Table 3).

## Discussion

With multivariate analysis considering 65 variables that include previously suggested AMD risk factors of systemic^10^,^11^,^12^,^13^, genetic^7^,^19^,^20^, and environmental factors, we found a strong effect of serum calcium level, *ARMS2* A69S genotype, age, and *C.* *pneumonia* infection on the development of drusen. While *ARMS2* genotype, age, *C.* *pneumonia* infection have previously been reported to be associated with AMD^1^,^21^,^22^,^23^, no study has reported the association between serum calcium level and AMD. Serum calcium levels are under tight hormonal control with a normal range of 8.6–10.3 mg/dL. Calcium plays a key role in membrane potential, which is important for muscle contraction, heart rate regulation, and nerve impulse generation. Hypocalcemia is caused by loss of calcium from, for example, renal failure, or insufficient entry of calcium into the circulation, due to hypoparathyroidism, magnesium depletion, etc. In the present study, we found that hypocalcemia was more frequent in large drusen cases than in controls (*P* = 0.0490). The reason for the association between low calcium level and AMD is not clear; however, the presence of calcium in drusen has been known as early as 1987 by using classic freeze-fracture and scanning electron microscope based elemental analysis^24^. Moreover, a recent study showed that calcium is present in very high concentration in drusen^25^. It has also been reported that basal ganglia calcification often occurs in idiopathic hypoparathyroidism patients and is correlated with hypocalcemia, choroid plexus calcification, and cataracts^26^. Although further studies are required, similar disease processes may affect calcification of the subretinal space, leading to the development of drusen, and a central nervous system with poor calcium control.

In the present study, we confirmed the association of age, *ARMS2* A69S genotype, HDL cholesterol, and hs-CRP levels with drusen, which were previously suggested as AMD risk factors^1^,^12^,^13^,^19^. However, multivariate analysis revealed that the effect size of hs-CRP and HDL cholesterol for drusen development was quite small (Fig. 1). These results are not surprising because several population-based cohort studies showed a lack of association between these factors and AMD^14^,^15^,^16^. Similarly, although α1-antitrypsin showed a potential association for drusen, the effect of this factor on disease development was limited (OR = 1.001).

*C. pneumonia* exposure has been suggested to be associated with AMD^21^,^22^,^23^. Kalayoglu et al reported that *C. pneumoniae* DNA was identified in surgically removed neovascular tissue from eyes with AMD^27^, but other studies, including a population-based study examining 3,654 adults, failed to find any association between *C. pneumoniae* antibody titers and AMD, thereby generating controversy^28^,^29^,^30^,^31^. However, in the present study assessing 60+ factors in 4,000+ adults, we found a strong effect of *C. pneumoniae* infection on drusen. To date, there is a consensus that the *CFH* gene is associated with advanced AMD^7^,^20^. CFH is known as the main soluble inhibitor of the alternative pathway, which prevents progression of the cascade by binding and inactivating complement component C3b^32^. Several complement system factors, their activators, and complement regulatory proteins were identified as cardinal constituents of drusen^8^,^33^ although the association between the *CFH* and early AMD remains controversial^34^,^35^. *C. pneumoniae* activates the alternative complement pathway or induces a chronic inflammatory state, which might contribute to the pathogenesis of AMD^21^,^22^,^23^. The present study, which showed a significant relationship between *C. pneumoniae* and the development of drusen, would indicate the significant role of the complement pathway in the inflammatory process with the disease development. On the other hand, we did not find strong associations between *CFH* genotypes and drusen. Taken together, our result would suggest that the activation of the alternative complement pathway by *C. pneumoniae* might be more important than that by *CFH* gene variation in the early stages of AMD in Asians.

Limitations of the present study include its cross-sectional design. In the present study, the role of previously suggested AMD risk factors, such as blood pressure^10^, BMI^11^, HDL cholesterol^12^, and hs-CRP^13^, were found to be limited. Also, we found no significant association between smoking and large drusen although Brinkman index of the large drusen group tended to be higher than that of controls (187.2 vs 173.2). These associations should be studied in a prospective study that evaluates multiple risk factors in AMD. Another limitation might be potential bias related to the high female to male ratio of the study subjects. However, a potential confounder in gender would be limited because a logistic regression analysis adjusting for gender was used in the present study. Further limitation is the lack of identifying a functional role of calcium in AMD. Although it is reported that calcium is present in very high concentration in drusen^25^, there might be subretinal deposits without calcification that are currently called “drusen” by fundoscopy. Also, it remains unknown if low calcium intake can be the risk of the development of drusen. Following basic and clinical research investigating the role of calcium in AMD is therefore needed.

In summary, by simultaneous evaluation of multiple factors including systemic, environmental, and genetic factors, we found a strong association between serum calcium level, *ARMS2* A69S genotype, age, and *C.* *pneumonia* infection and the development of drusen. Our findings suggest a significant role for these factors during the early course of AMD.

## Methods

### The Nagahama Study Population

Participants were part of a study that was ancillary to the Nagahama Study, the details of which have been reported elsewhere^6^. The Nagahama Study is a community-based prospective cohort study designed to determine the prevalence and risk factors of various diseases in a community. Between November 2008 and November 2010, a total of 6,118 residents of Nagahama City aged ≥50 years participated in the Nagahama Study. All protocols and informed consent procedures were approved by the Kyoto University Graduate School and Faculty of Medicine Ethics Committee, the Ad Hoc Review Board of the Nagahama Cohort Project, and the Nagahama Municipal Review Board of Personal Information Protection. This study was carried out in accordance with the approved guidelines.

At baseline, all participants were asked to undergo eye examinations including automatic refractometry (Autorefractor ARK-530; Nidek Co Ltd, Aichi, Japan), axial length measurements (IOL Master; Carl Zeiss AG, Oberkochen, Germany), and fundus photography using a digital retinal camera (CR-DG10; Canon Inc., Tokyo, Japan) in a darkened room. All study subjects (n = 5,595) satisfied the following criteria: (a) age ≥50 years; (b) nonmydriatic fundus photographs for both eyes of sufficient quality for grading lesions; and (c) no other retinal diseases that would affect the precise grading of a macular lesion (such as diabetic retinopathy, retinal vein occlusion, or epiretinal membranes)^6^. Each fundus photograph was graded twice by two independent ophthalmologists for phenotypes of AMD through standardized grading procedures^3^,^6^. For the sake of multivariate analysis, we selected cases and controls from the study subjects. Based on the grading procedure, 971 individuals who had a large drusen (soft distinct and soft indistinct drusen ≥125 µm in diameter) in either eye were included as the study cases^36^,^37^. As controls, 3,209 individuals lacking any sign of AMD (drusen, retinal pigment epithelial abnormalities, or advanced AMD) in both eyes were selected. Twenty-nine cases with advanced AMD and a total of 1,386 individuals, including 276 with pigment epithelial abnormalities only and 1,110 with drusen less than 125 µm in diameter or reticular pseudodrusen, were excluded from the analysis.

### Systemic Factor Analysis

A total of 60 systemic factors, including physical examination, hematological tests, biochemical tests, urinalysis, immunological tests, endocrinological tests, and allergy tests, were analyzed in each subject (Table S1). Blood and urine collection and processing were performed according to a standard protocol (XE-2100 hematology analyzer; Sysmex Co Ltd, Hyogo, Japan and LABOSPECT 008 Hitachi automatic analyzer; Hitachi Ltd, Tokyo, Japan). When the result exceeded the detection range, it was examined using a dilution test. The results of allergy tests were categorized as class 0 to 6 (class 0 = no allergy and class 6 = severe allergy) in a standard manner. To assess the environmental effect, information on smoking status was obtained via a self-report questionnaire. Total smoking amount was ascertained using the Brinkman index^38^, calculated as the daily number of cigarettes × years of smoking.

### Genotyping

To assess the role of genetic factors for disease, genomic DNA was prepared from the peripheral blood of 4,201 subjects. The most robust AMD-associated variants, A69S (rs10490924) on *ARMS2*^19^ and Y402H (rs1061170) and I62V (rs800292) on *CFH*^7^,^20^, were genotyped using TaqMan single nucleotide polymorphism assay with the PRISM 7700 system (Applied Biosystems, Inc., Foster City, CA, USA) and Human610-Quad BeadChips and HumanOmni2.5 BeadChips (Illumina, Inc., San Diego, CA, USA).

### Statistical Analysis

Descriptive statistics are presented, and estimates of center and dispersion are described as mean and standard deviation (SD). To compare demographic characteristics, analysis of variance (ANOVA) or the χ^2^ test were used.

For multivariate analysis, independent factors associated with drusen were determined using logistic regression. At first, univariate analyses were conducted to screen independent variables for a potential association with drusen. Since correlated variables should not be entered together in the same multivariable model, collinear variables were excluded from the analyses; for example, the variable for immunoglobulin A (IgA) antibodies for *Chlamydia pneumoniae* was excluded in lieu of the immunoglobulin G (IgG) antibodies for *C. pneumoniae*. Variables with *P* < 0.25 in the univariate analysis were entered in the multivariable analysis along with other predictors previously reported as being significantly associated with AMD (e.g., blood pressure^10^, body mass index [BMI]^11^, high-density lipoprotein [HDL] cholesterol^12^, high-sensitivity C-reactive protein [hs-CRP]^13^, smoking status, and the genetic factors). The final multivariate model was built through stepwise selection using a backward-elimination approach. Software R (http://www.r-project.org/) was used for statistical analyses. *P* values < 0.05 were considered statistically significant.

## Author Contributions

I.N., K.Y., F.M. and N.Y. have designed the study. I.N., K.Y., H.N., Y.A-K., M.M., F.M. and NSG acquired the data. I.N., T.K. and R.Y. analyzed and interpreted data. K.Y., A.T. and N.Y. supervised the study. I.N. wrote the manuscript. All authors reviewed the manuscript.

## Additional information

The list of authors in the Nagahama Study Group: T.N., A.S., S.K., Y.T., and M.M.

## Supplementary Material

Supplementary Information Supplementary Information

## Figures and Tables

**Table 1:** Predominant Characteristics of the Study Subjects

	Large drusen	Control	*P*-value
N	971	3209	
Age, y	65.1 ± 5.9	61.7 ± 6.5	< 0.001
Sex, n (%)			
Male	357 (36.8)	1121 (34.9)	0.295
Female	614 (63.2)	2088 (65.1)	
Smoking, n (%)			
Never	646 (66.5)	2127 (66.3)	0.856
Past	201 (20.7)	687 (21.4)	
Current	124 (12.8)	395 (12.3)	
Brinkman index*	187.2 ± 360.7	173.2 ± 343.8	0.272
*ARMS2* A69S, n (%)			
GG	239 (35.0)	1065 (43.5)	< 0.001
GT	334 (49.0)	1102 (45.0)	
TT	109 (16.0)	284 (11.6)	
*CFH* Y402H, n (%)			
CC	591 (87.2)	2122 (86.8)	0.908
CT	81 (11.9)	315 (12.9)	
TT	6 (0.9)	8 (0.3)	
*CFH* I62V, n (%)			
GG	241 (35.3)	927 (37.8)	0.147
GA	328 (48.1)	1163 (47.5)	
AA	113 (16.6)	361 (14.7)	

*The Brinkman index was calculated as daily number of cigarettes × years smoking.

**Table 2:** Summary of the Multivariate Analyses for Large Drusen

	Beta	SE	OR (95%CI)	*P*-value
Age	0.013	0.001	1.013 (1.011–1.015)	< 0.001
*ARMS2* A69S genotype	0.045	0.011	1.046 (1.025–1.069)	< 0.001
Calcium	−0.070	0.021	0.932 (0.894–0.972)	1.05 × 10^−3^
HDL cholesterol	0.001	<0.001	1.001 (1.000–1.002)	2.07 × 10^−3^
α1-Antitrypsin	0.001	<0.001	1.001 (1.000–1.002)	0.0268
Hs-CRP	<0.001	<0.001	1.000 (1.000–1.000)	0.0369
*Chlamydia pneumoniae* antibody, IgG	0.020	0.010	1.020 (1.001–1.040)	0.0440
*CFH* I62V genotype	0.017	0.010	1.017 (0.997–1.039)	0.100
Weed pollens allergy specific IgE	−0.016	0.010	0.984 (0.964–1.004)	0.113

SE, standard error; OR, odds ratio; HDL, high-density lipoprotein; Hs-CRP, high-sensitivity C-reactive protein; IgG, immunoglobulin G; IgE, immunoglobulin E.

**Table 3:** Status of the Serum Calcium Level and Chlamydia *Pneumoniae* infection in the Study Group

	Large drusen	Control	P-value
N	971	3209	
Serum calcium level (mg/dL)			
<8.6 (Hypocalcemia)	70 (7.2%)	177 (5.5%)	0.049
8.6–10.3 (Normal)	900 (92.8%)	3028 (94.4%)	0.0702
>10.3 (Hypercalcemia)	0 (0.0%)	4 (0.1%)	0.271
C. Pneumoniae IgG			
>1.1 (Positive infection)	548 (56.4%)	1659 (51.7%)	0.00956
0.9–1.1 (Equivocal)	109 (11.2%)	360 (11.2%)	0.995
<0.9 (Negative infection)	314 (32.4%)	1190 (37.1%)	0.00694

## References

[b1] de Jong P. T. Age-related macular degeneration. N Engl J Med 355, 1474–85 (2006). 17021323 10.1056/NEJMra062326

[b2] Hogg R. E. & Chakravarthy U. Visual function and dysfunction in early and late age-related maculopathy. Prog Retin Eye Res 25, 249–76 (2006). 16580242 10.1016/j.preteyeres.2005.11.002

[b3] Ferris F. L. *et al.* A simplified severity scale for age-related macular degeneration: AREDS Report No. 18. Arch Ophthalmol 123, 1570–4 (2005). 16286620 10.1001/archopht.123.11.1570 PMC1473206

[b4] Day S., Acquah K., Lee P. P., Mruthyunjaya P. & Sloan F. A. Medicare costs for neovascular age-related macular degeneration, 1994-2007. Am J Ophthalmol 152, 1014–20 (2011). 21843875 10.1016/j.ajo.2011.05.008 PMC3219793

[b5] Smith W., Mitchell P. & Leeder S. R. Smoking and age-related maculopathy. The Blue Mountains Eye Study. Arch Ophthalmol 114, 1518–23 (1996). 8953988 10.1001/archopht.1996.01100140716016

[b6] Nakata I. *et al.* Prevalence and characteristics of age-related macular degeneration in the Japanese population: the nagahama study. Am J Ophthalmol 156, 1002–1009.e2 (2013). 23938127 10.1016/j.ajo.2013.06.007

[b7] Hageman G. S. *et al.* A common haplotype in the complement regulatory gene factor H (HF1/CFH) predisposes individuals to age-related macular degeneration. Proc Natl Acad Sci U S A 102, 7227–32 (2005). 15870199 10.1073/pnas.0501536102 PMC1088171

[b8] Gold B. *et al.* Variation in factor B (BF) and complement component 2 (C2) genes is associated with age-related macular degeneration. Nat Genet 38, 458–62 (2006). 16518403 10.1038/ng1750 PMC2921703

[b9] Nakata I. *et al.* Significance of C2/CFB Variants in Age-Related Macular Degeneration and Polypoidal Choroidal Vasculopathy in a Japanese Population. Invest Ophthalmol Vis Sci 53, 794–8 (2012). 22232432 10.1167/iovs.11-8468

[b10] Hyman L., Schachat A. P., He Q. & Leske M. C. Hypertension, cardiovascular disease, and age-related macular degeneration. Age-Related Macular Degeneration Risk Factors Study Group. Arch Ophthalmol 118, 351–8 (2000). 10721957 10.1001/archopht.118.3.351

[b11] Seddon J. M., Cote J., Davis N. & Rosner B. Progression of age-related macular degeneration: association with body mass index, waist circumference, and waist-hip ratio. Arch Ophthalmol 121, 785–92 (2003). 12796248 10.1001/archopht.121.6.785

[b12] Tan J. S., Mitchell P., Smith W. & Wang J. J. Cardiovascular risk factors and the long-term incidence of age-related macular degeneration: the Blue Mountains Eye Study. Ophthalmology 114, 1143–50 (2007). 17275090 10.1016/j.ophtha.2006.09.033

[b13] Boekhoorn S. S., Vingerling J. R., Witteman J. C., Hofman A. & de Jong P. T. C-reactive protein level and risk of aging macula disorder: The Rotterdam Study. Arch Ophthalmol 125, 1396–401 (2007). 17923549 10.1001/archopht.125.10.1396

[b14] Klein R. *et al.* Early age-related maculopathy in the cardiovascular health study. Ophthalmology 110, 25–33 (2003). 12511342 10.1016/s0161-6420(02)01565-8

[b15] Tomany S. C. *et al.* Risk factors for incident age-related macular degeneration: pooled findings from 3 continents. Ophthalmology 111, 1280–7 (2004). 15234127 10.1016/j.ophtha.2003.11.010

[b16] Buch H. *et al.* Risk factors for age-related maculopathy in a 14-year follow-up study: the Copenhagen City Eye Study. Acta Ophthalmol Scand 83, 409–18 (2005). 16029262 10.1111/j.1600-0420.2005.00492.x

[b17] Park J. H. *et al.* Distribution of allele frequencies and effect sizes and their interrelationships for common genetic susceptibility variants. Proc Natl Acad Sci U S A 108, 18026–31 (2011). 22003128 10.1073/pnas.1114759108 PMC3207674

[b18] Klein R., Klein B. E., Jensen S. C. & Meuer S. M. The five-year incidence and progression of age-related maculopathy: the Beaver Dam Eye Study. Ophthalmology 104, 7–21 (1997). 9022098 10.1016/s0161-6420(97)30368-6

[b19] Jakobsdottir J. *et al.* Susceptibility genes for age-related maculopathy on chromosome 10q26. Am J Hum Genet 77, 389–407 (2005). 16080115 10.1086/444437 PMC1226205

[b20] Hayashi H. *et al.* CFH and ARMS2 variations in age-related macular degeneration, polypoidal choroidal vasculopathy, and retinal angiomatous proliferation. Invest Ophthalmol Vis Sci 51, 5914–9 (2010). 20574013 10.1167/iovs.10-5554

[b21] Kalayoglu M. V., Galvan C., Mahdi O. S., Byrne G. I. & Mansour S. Serological association between Chlamydia pneumoniae infection and age-related macular degeneration. Arch Ophthalmol 121, 478–82 (2003). 12695244 10.1001/archopht.121.4.478

[b22] Ishida O. *et al.* Is Chlamydia pneumoniae infection a risk factor for age related macular degeneration? Br J Ophthalmol 87, 523–4 (2003). 12714382 10.1136/bjo.87.5.523 PMC1771658

[b23] Robman L. *et al.* Exposure to Chlamydia pneumoniae infection and progression of age-related macular degeneration. Am J Epidemiol 161, 1013–9 (2005). 15901621 10.1093/aje/kwi130

[b24] Ulshafer R. J., Allen C. B., Nicolaissen B. & Rubin M. L. Scanning electron microscopy of human drusen. Invest Ophthalmol Vis Sci 28, 683–9 (1987). 2435670

[b25] Flinn J. M., Kakalec P., Tappero R., Jones B. & Lengyel I. Correlations in distribution and concentration of calcium, copper and iron with zinc in isolated extracellular deposits associated with age-related macular degeneration. Metallomics 6, 1223–8. (2014). 24740686 10.1039/c4mt00058g

[b26] Goswami R. *et al.* Prevalence and progression of basal ganglia calcification and its pathogenic mechanism in patients with idiopathic hypoparathyroidism. Clin Endocrinol (Oxf) 77, 200–6 (2012). 22288727 10.1111/j.1365-2265.2012.04353.x

[b27] Kalayoglu M. V. *et al.* Identification of Chlamydia pneumoniae within human choroidal neovascular membranes secondary to age-related macular degeneration. Graefes Arch Clin Exp Ophthalmol 243, 1080–90 (2005). 15909160 10.1007/s00417-005-1169-y

[b28] Robman L. *et al.* Exposure to Chlamydia pneumoniae infection and age-related macular degeneration: the Blue Mountains Eye Study. Invest Ophthalmol Vis Sci 48, 4007–11 (2007). 17724180 10.1167/iovs.06-1434

[b29] Miller D. M. *et al.* The association of prior cytomegalovirus infection with neovascular age-related macular degeneration. Am J Ophthalmol 138, 323–8 (2004). 15364212 10.1016/j.ajo.2004.03.018

[b30] Haas P. *et al.* Complement factor H gene polymorphisms and Chlamydia pneumoniae infection in age-related macular degeneration. Eye (Lond) 23, 2228–32 (2009). 19169230 10.1038/eye.2008.422 PMC4853919

[b31] Klein R. *et al.* Systemic markers of inflammation, endothelial dysfunction, and age-related maculopathy. Am J Ophthalmol 140, 35–44 (2005). 15939388 10.1016/j.ajo.2005.01.051

[b32] Despriet D. D. *et al.* Complement factor H polymorphism, complement activators, and risk of age-related macular degeneration. JAMA 296, 301–9 (2006). 16849663 10.1001/jama.296.3.301

[b33] Anderson D. H., Mullins R. F., Hageman G. S. & Johnson L. V. A role for local inflammation in the formation of drusen in the aging eye. Am J Ophthalmol 134, 411–31 (2002). 12208254 10.1016/s0002-9394(02)01624-0

[b34] Chen J. H. *et al.* No association of age-related maculopathy susceptibility protein 2/HtrA serine peptidase 1 or complement factor H polymorphisms with early age-related maculopathy in a Chinese cohort. Mol Vis 19, 944–54 (2013). 23687431 PMC3654849

[b35] Holliday E. G. *et al.* Insights into the genetic architecture of early stage age-related macular degeneration: a genome-wide association study meta-analysis. PLoS One 8, e53830 (2013). 23326517 10.1371/journal.pone.0053830 PMC3543264

[b36] Oshima Y. *et al.* Prevalence of age related maculopathy in a representative Japanese population: the Hisayama study. Br J Ophthalmol 85, 1153–7 (2001). 11567955 10.1136/bjo.85.10.1153 PMC1723746

[b37] Yasuda M. *et al.* Nine-year incidence and risk factors for age-related macular degeneration in a defined Japanese population the Hisayama study. Ophthalmology 116, 2135–40 (2009). 19744734 10.1016/j.ophtha.2009.04.017

[b38] Brinkman G. L. & Coates E. O. The effect of bronchitis, smoking, and occupation on ventilation. Am Rev Respir Dis 87, 684–93 (1963). 14015517 10.1164/arrd.1963.87.5.684

