# Supplementary material for: Calcium, ARMS2 Genotype, and Chlamydia Pneumoniae Infection in Early Age-Related Macular Degeneration: a Multivariate Analysis from the Nagahama Study
Source: Sci Rep. 2015 Mar 20;5:9345. doi: 10.1038/srep09345 (PMC4366853; doi:10.1038/srep09345)
Supplement: Supplementary Information [file srep09345-s1.pdf]

Calcium, *ARMS2* Genotype, and *Chlamydia Pneumoniae* Infection in Early Age-Related Macular Degeneration: a Multivariate Analysis from the Nagahama Study

Isao Nakata,[1,2] Kenji Yamashiro,*[1] Takahisa Kawaguchi,[2] Hideo Nakanishi,[1,2] Yumiko Akagi-Kurashige,[1,2] Masahiro Miyake,[1,2] Akitaka Tsujikawa,[1] the Nagahama Study Group, Ryo Yamada,[2] Fumihiko Matsuda,[2] Nagahisa Yoshimura.[1]

Author affiliations:

[1]Department of Ophthalmology, Kyoto University Graduate School of Medicine, Kyoto, Japan

[2]Center for Genomic Medicine/Inserm U.852, Kyoto University Graduate School of Medicine, Kyoto, Japan

Table S1. Genetic and Systemic Factors Analyzed in the Study Subjects

Examination Field	Examination Item (unit)	Examination Field	Examination Item (unit)
Physical	Age (year)	Urinalysis	Urine specific gravity
	Sex		Urine Na (mEq/L)
	Height (cm)		Urine K (mEq/L)
	Weight (kg)		Urine creatinine (mg/dL)
	Body mass index		Urine microalbumin (ug/min)
	Abdominal circumference (cm)	Tumor-associated	Free prostate-specific antigen (ng/mL)
	Systolic blood pressure (mmHg)	Immunological	α1-Antitrypsin (mg/dL)
	Diastolic blood pressure (mmHg)		High-sensitivity C-reactive protein (ng/dL)
Hematological	White blood cell count (x100/uL)		Anti-citrullinated peptide antibody (U/mL)
	Red blood cell count (x10000/uL)		*Mycoplasma pneumonia*
	Hemoglobin (g/dL)		Rheumatoid factor (IU/mL)
	Hematocrit (%)		Antinuclear antibody
	Platelet (x10000/uL)		*Chlamydia pneumoniae* IgG
Biochemical	Serum aspartate aminotransferase (IU/L)		*Chlamydia pneumoniae* IgA
	Alanine aminotransferase (IU/L)		IgE (IgE nonspecific)
	γ-Glutamyl transpeptidase (IU/L)	Endocrinological	B-type natriuretic peptide (pg/mL)
	Total cholesterol (mg/dL)		N-telopeptides crosslinks (nmol BCE/L)
	Neutral fat (mg/dL)		C-telopeptides crosslinks (ug/L)
	High-density lipoprotein cholesterol (mg/dL)		Insulin (uIU/mL)
	Creatinine (mg/dL)	Allergy specific IgE	Poaceae (class)
	Albumin (g/dL)		Mold (class)
	Blood glucose (mg/dL)		Weed pollen (class)
	Glycated hemoglobin (mg/dL)		House dust (class)
	Low-density lipoprotein cholesterol (mg/dL)		*Dermatophagoides pteronyssinus* (class)
	Choline esterase (IU/L)		Cedar pollen (class)
	Total protein (g/dL)		Cat dander (class)
	Free fatty acid (mEq/L)		Dog dander (class)
	Calcium (mg/dL)	Self-report questionnaire	Smoking status
	Alkaline phosphatase (IU/L)		Brinkman index
	Inorganic phosphorus (mg/dL)	Genotype	*ARMS2* A69S, rs10490924
	Total bilirubin (mg/dL)		*CFH* Y402H, rs1061170
	Cotinine		*CFH* I62V, rs800292
	Angiotensin-converting enzyme (U/L)		

BCE, Bone collagen equivalents.

Table S2. Summary of the Univariate Analyses for Large Drusen.

	P Value
Age (year)	< 0.001
α1-Antitrypsin (mg/dL)	< 0.001
ARMS2 A69S, rs10490924	< 0.001
Cedar pollen allergy (class)	< 0.001
Calcium (mg/dL)	< 0.001
Dog dander allergy (class)	< 0.001
Platelet (×10000/uL)	2.1×10^{-3}
D pteronyssinus allergy (class)	2.9×10^{-3}
B-type natriuretic peptide (pg/mL)	3.3×10^{-3}
Chlamydia pneumoniae IgG	5.0×10^{-3}
Low-density lipoprotein cholesterol (mg/dL)	5.3×10^{-3}
Albumin (g/dL)	0.0104
Height (cm)	0.0113
House dust allergy (class)	0.0175
Total cholesterol (mg/dL)	0.0279
Cat dander allergy (class)	0.0333
N-telopeptides crosslinks (nmol BCE/L)	0.0345
Poaceae allergy (class)	0.0349
Weight (kg)	0.0388
Choline esterase (IU/L)	0.0429
Urine creatinine (mg/dL)	0.0475
Weed pollen allergy (class)	0.0555
Inorganic phosphorus (mg/dL)	0.0821
Systolic blood pressure (mmHg)	0.137
CFH I62V, rs800292	0.147
Diastolic blood pressure (mmHg)	0.178
Alkaline phosphatase (IU/L)	0.182
Neutral fat (mg/dL)	0.186
Red blood cell count (×10000/uL)	0.223
